# Longitudinal multi-omics alterations response to 8-week risperidone monotherapy: Evidence linking cortical thickness, transcriptomics and epigenetics

**DOI:** 10.3389/fpsyt.2023.1127353

**Published:** 2023-03-02

**Authors:** Xiaofen Zong, Gaohua Wang, Zhaowen Nie, Simeng Ma, Lijun Kang, Nan Zhang, Shenhong Weng, Qing Tan, Junjie Zheng, Maolin Hu

**Affiliations:** ^1^Department of Psychiatry, Renmin Hospital of Wuhan University, Wuhan, Hubei, China; ^2^School of Mathematics and Statistics, Wuhan University, Wuhan, Hubei, China; ^3^Hubei Key Laboratory of Computational Science, Wuhan University, Wuhan, Hubei, China; ^4^The Early Intervention Unit, Department of Psychiatry, Affiliated Nanjing Brain Hospital, Nanjing Medical University, Nanjing, Jiangsu, China; ^5^The Functional Brain Imaging Institute, Nanjing Medical University, Nanjing, Jiangsu, China; ^6^Department of Psychiatry, The Second Xiangya Hospital of Central South University, Changsha, Hunan, China; ^7^Department of Psychiatry, Henan Mental Hospital, The Second Affiliated Hospital of Xinxiang Medical University, Xinxiang, Henan, China

**Keywords:** Allen Human Brain Atlas, risperidone, schizophrenia, magnetic resonance imaging, cortical thickness, DNA methylation

## Abstract

**Background:**

Antipsychotic treatment-related alterations of cortical thickness (CT) and clinical symptoms have been previously corroborated, but less is known about whether the changes are driven by gene expression and epigenetic modifications.

**Methods:**

Utilizing a prospective design, we recruited 42 treatment-naive first-episode schizophrenia patients (FESP) and 38 healthy controls. Patients were scanned by TI weighted imaging before and after 8-week risperidone monotherapy. CT estimation was automatically performed with the FreeSurfer software package. Participants' peripheral blood genomic DNA methylation (DNAm) status, quantified by using Infinium^®^ Human Methylation 450K BeadChip, was examined in parallel with T1 scanning. In total, CT measures from 118 subjects and genomic DNAm status from 114 subjects were finally collected. Partial least squares (PLS) regression was used to detect the spatial associations between longitudinal CT variations after treatment and cortical transcriptomic data acquired from the Allen Human Brain Atlas. Canonical correlation analysis (CCA) was then performed to identify multivariate associations between DNAm of PLS1 genes and patients' clinical improvement.

**Results:**

We detected the significant PLS1 component (2,098 genes) related to longitudinal alterations of CT, and the PLS1 genes were significantly enriched in neurobiological processes, and dopaminergic- and cancer-related pathways. Combining Laplacian score and CCA analysis, we further linked DNAm of 33 representative genes from the 2,098 PLS1 genes with patients' reduction rate of clinical symptoms.

**Conclusions:**

This study firstly revealed that changes of CT and clinical behaviors after treatment may be transcriptionally and epigenetically underlied. We define a “three-step” roadmap which represents a vital step toward the exploration of treatment- and treatment response-related biomarkers on the basis of multiple omics rather than a single omics type as a strategy for advancing precise care.

## Introduction

Cortical thickness (CT) relates to the number of neurons and the neuropil within a cohort of cortical neurons originating from a single neuronal progenitor (1). Measurement of CT may therefore allow probing changes in brain maturation and growth, which are increasingly being proposed as neuropathological mechanisms of schizophrenia. CT mapping in this disease has indeed shown abnormalities of the cortical areas, particularly in parts of the temporal and frontal lobes (2). Antipsychotics are commonly used as first-line regimens for the treatment of schizophrenia, as well as bipolar disorders. Noteworthily, accumulating evidence supports that antipsychotic agents may affect CT in schizophrenia patients, inducing excessive thinning widely distributed in most cortical areas (3–7), which has raised concerns about the potential neurotoxic side effects of antipsychotic agents that might need to be taken into consideration when prescribing them.

Although antipsychotic therapy may have potential contribution to cortical morphological alterations, less is known about whether treatment-related changes of CT after treatment are driven by genetic factors. Recent brain expression atlases, bridge the gap between genetic factors and brain morphology phenotypes. The Allen Human Brain Atlas (AHBA), a publicly available transcriptomic dataset (8), has been utilized to identify transcriptomic signatures associated with brain structural alterations of individuals with mental disorders, such as schizophrenia and major depressive disorder (MDD) (9, 10), which uncovers the molecular foundation of regional brain vulnerability to major mental disorders. By using the transcriptomic dataset of AHBA, researchers have uncovered the molecular foundation of regional brain vulnerability to major mental disorders (10). However, less is known about whether CT may be specifically related to co-expression of a set of genes relevant to neurobiological processes.

Epigenetic modifications, some heritable changes that are not due to alterations in DNA sequence, mediate between gene expression and environmental insults, and then alter and stably maintain gene expression levels (11). DNA methylation (DNAm), among the epigenetic modifications, is a stable epigenetic mechanism. Recent research on psychiatric disorders has only begun to investigate the associations between DNAm alterations and antipsychotic therapy outcomes (12). Combining of brain anatomical phenotypes, brain gene expression and DNAm is important for developing treatment- and treatment outcome-related biomarkers, as it can integrate multi-omics data to comprehensively explain heterogeneity of treatment and treatment response across individuals, although few researches have been conducted recently. Noteworthily, although DNAm status is reported to be tissue-specific, 10.9% CpG sites were strongly (*r* > 0.5) associated between brain tissues and peripheral blood cells (13), implying that DNAm of peripheral blood cells may play as a surrogate for DNAm levels of the brain.

This study firstly integrates multi-omics data including peripheral DNAm, AHBA transcriptomic data and CT to comprehensively explain the transcriptional and epigenetically basis underlying CT alterations and clinical symptom improvement after acute antipsychotic treatment. Patients were given risperidone monotherapy for 8 weeks to control for the confounding effects of multidrug therapy on treatment response. CT estimation was automatically performed with the FreeSurfer software package. Partial least squares (PLS) regression was used to detect the spatial associations between longitudinal CT variations after treatment and cortical transcriptomic data acquired from the AHBA. Patients' peripheral blood genomic DNAm, examined in parallel with T1 scanning at the two time points, was quantified by using Infinium^®^ Human Methylation 450K BeadChip. We then performed Canonical Correlation Analysis (CCA) to identify multivariate associations between patients' clinical symptom improvement and their longitudinal alterations of DNAm of PLS1 genes. On the basis of previous evidence, it was hypothesized that: (1) alterations of CT and clinical symptoms after antipsychotic treatment would be transcriptionally and epigenetically underlied; (2) phenotypic variations of CT after treatment would be spatially correlated with brain gene expression acquired from the AHBA, and these genes would be enriched in dopaminergic-related pathways and neurobiological processes; (3) the DNAm levels of part of PLS1 genes would relate to patients' clinical response.

## Methods

### Participants

We recruited 42 drug-naive patients with first-episode schizophrenia (Patients-0W) and their matched healthy controls (*n* = 38) in Henan Mental Hospital (China) from 12/2012 to 01/2014. This dataset has been utilized by our group (14–16). All patients were diagnosed by experienced psychiatrists based on the Structured Clinical Interview for DSM-IV-TR. Patients' disease duration was <12 months. SCID-non-patient edition was used to scan controls to ensure that they have no history of other mental disorders or neurological diseases.

The study was performed following the Declaration of Helsinki. It was approved by Ethics Committees of the Henan Mental Hospital, Xinxiang, China and the Second Xiangya Hospital of Central South University, Changsha China (protocol code S088, 2012). All subjects in this study provided written informed consent.

### Antipsychotic therapy

The starting dose of risperidone was 1 or 2 mg/day, and then slowly titrated after 1 week. Specifically, we increased its dosage at 1-week interval until the patients had obvious clinical improvement. For those individuals who did not show satisfactory improvement after 4 weeks, they were permitted to reach a maximum daily dose of 6 mg/day. All 42 patients were prescribed with risperidone for 8 weeks, and not allowed to take mood stabilizers or antidepressants. The therapeutic safety was assessed weekly through clinical interviews.

### Psychopathological assessments

Patients' symptomatic severity was assessed both at baseline and after 8-week treatment by using the Positive and Negative Syndrome Scale (PANSS) (17), which consists of general psychopathology (PANSS-G, 16 items, G1–G16), negative (PANSS-N, 7 items, N1–N7), and positive (PANSS-P, 7 items, P1–P7). The improvement in each symptom dimension was measured by the reduction rate (18). Reduction rates for positive, negative and general psychopathology symptoms were respectively equal to PANSS-P_0W—PANSS-P_8W/PANSS-P_0W-7, PANSS-N_0W—PANSS-N_8W/PANSS-N_0W-7, as well as PANSS-G_0W—PANSS-G_8W/PANSS-G_0W-16.

## “Three-steps” analysis detecting longitudinal multi-omics alterations response to treatment

**Figure 1:**
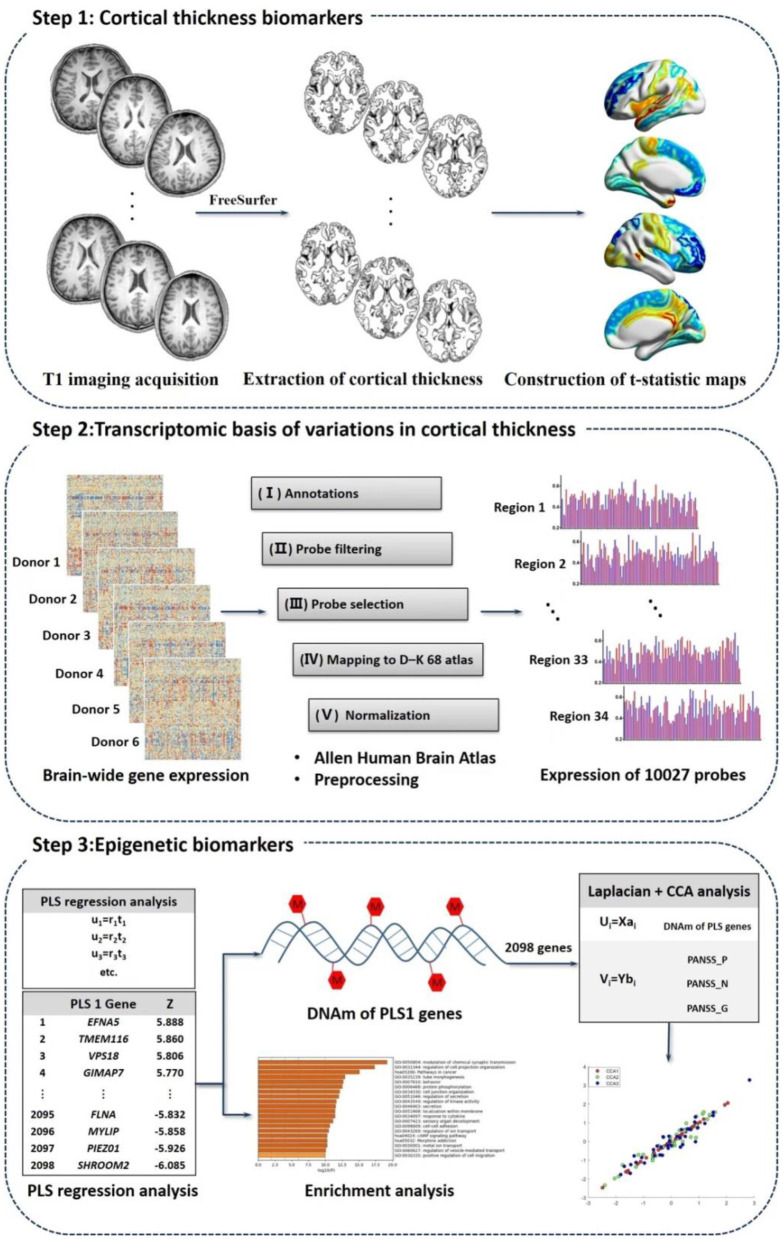
Study overview of imaging-transcriptomics-epigenetics analysis.

We explored biomarkers correlated to treatment, treatment response as well as schizophrenia pathology using multi-omics measures linking CT measures, transcriptomic signatures and peripheral DNAm values based on the following steps (Figure 1).

## Step 1: CT biomarkers related to schizophrenia and treatment

### T1 imaging acquisition

The participants underwent T1 weighted images on a 3.0T Siemens MRI scanner (Vero) at the Magnetic Imaging Center, the Henan Mental Hospital, Xinxiang, China. Patients were scanned both before and after 8-week treatment, while healthy controls only underwent the baseline neuroimaging assessment. Detailed MRI parameters were shown in the Supplementary material. There were 4 patients withdrawing the follow-up T1 scans. We therefore obtained imaging data of 42 Patients_0W, 38 patients after treatment (Patients_8W), and 38 healthy volunteers.

### Image analysis

CT estimation was automatically processed with the FreeSurfer software package (v5.3.0; http://surfer.nmr.mgh.harvard.edu/) according to the programme segmentation procedure, the technical details of which were described in the prior publication (19).

### Quality control of T1W data

To check the differences of image quality and head motion among the three groups, we computed the Euler number (20) for each MRI image. This quality control method was proposed as a way to quantitatively evaluate image quality (20–22). We then respectively used independent and paired samples *t*-test to compare the case-control and longitudinal differences of Euler number. We detected no significant case-control or longitudinal differences in the Euler number (*Ps* > 0.05).

### Extraction of CT

The cerebral cortex were subdivided into 68 areas (34 per hemisphere) following the Desikan–Killiany–Tourville (DKT) cortical labeling protocol (23). Each of the 68 regions was defined by the automated FreeSurfer segmentation procedure. We also extracted Total intracranial volume (TIV) to adjust for the confounding factors of brain sizes.

### Construction of *t*-statistic maps

All the CT measures of 68 regions were residualized with respect to TIV, sex and age utilizing generalized linear models before performing between-group comparisons. We respectively used independent samples *t*-test and paired samples *t*-test to compare the case-control and longitudinal differences in CT measures, and then obtained case-control and longitudinal *t*-statistic maps.

## Step 2: Transcriptomic basis of variations in CT related to schizophrenia and treatment

### Preprocessing of AHBA data

Transcriptional profiles, consisting of expression measures of 20,737 genes (from 58,692 probes), were acquired from the public database AHBA (http://www.brain-map.org) (8). The detailed information about AHBA was shown in Supplementary material. We preprocessed the transcriptional data according to previously published 5 steps (24): (1) annotations from probes to genes; (2) filtering probes; (3) screening representative probes; (4) assigning DKT-68 atlas; (5) normalizing expression levels. The preprocessing related codes are available at github (details see https://github.com/BMHLab/AHBAprocessing respectively). After preprocessing, we obtained 10,027 probes in each brain sample. Not all donors have samples in the right hemisphere (24), we therefore did not analyze tissue samples in the right hemisphere.

### PLS regression analysis

We respectively coregistered the two *t*-statistic maps (left hemisphere) with transcriptional maps (brain tissues of the left hemisphere in the AHBA) according to the DKT-68 atlas. Partial least squares (PLS) regression (9, 21) was then conducted to evaluate the spatial correlations between transcriptional scores and neuroimaging *t*-statistic maps. In the PLS analysis, the *t*-statistic map (for 34 brain regions) was set as response variables, and expression data of the 10,027 probes for the 34 brain areas were predictor variables. The first component (PLS1) obtained in the PLS analysis was the linear combination of transcriptional profiles significantly related to the *t*-statistic map. We conducted Permutation test (1,000 times) to test the null hypothesis that the PLS1 explained no more covariance between *t*-statistic maps and gene expression levels than expected by chance. We then performed Bootstrapping (500 bootstrap samples) to measure the weight of each PLS1 gene. The ratio of each region's weight to its standard error in the bootstrap was assessed as the *Z*-values. The normalized weights of each PLS1 gene were ranked through one-sample *Z*-tests. After FDR correction, we obtained gene lists (with *P*_*FDR*_ < 0.05) contributing to the PLS1.

### Enrichment analysis

We performed enrichment analysis for the above detected PLS1 genes with |*Z*| > 3 (21) (all FDR < 0.05), using an online tool Metascape (https://metascape.org), which provides automated meta-analysis to obtain enriched biological processes (gene ontology, GO) and pathways (Kyoto encyclopedia of genes and genomes, KEGG) (25). We filtered enrichment terms to those pathways and biological processes that met *P*_*FDR*_ < 0.01.

## Step 3: Epigenetic biomarkers

### DNAm of PLS1 genes

The peripheral blood DNAm status in both Patients_8W (*n* = 38) and Patients_0W (*n* = 38) was evaluated in parallel with T1W scanning. Genomic DNAm values were measured in the above samples by using Illumina 450K Genechip. The epigenetic dataset has been used previously by our group (15, 16). The average value of all the CpG sites in each PLS1 gene was used to represent the DNAm level of this gene. We demonstrated detailed information about DNA extraction, quality control (QC), bisulfite conversion, Genechip analysis, microarray data processing in Supplementary material.

### CCA analysis

Canonical correlation analysis (CCA) (26), a data-driven analysis among datasets each consisting of multiple variables, was utilized to identify multivariate associations between DNAm of PLS1 genes and clinical data. CCA aims to detect optimal linear combinations of multivariate epigenetic and behavioral measures by maximally relating the DNAm levels to clinical variables. The CCA analysis was performed as follows:


$$\begin{array}{l} U_{i}=Xa_{i} \end{array}$$

(1) $$\begin{array}{l} V_{i}=Yb_{i}, \end{array}$$

where *X* is the selected gene features by Laplacian score importance, and *Y* is the clinical behaviors (PANSS scores). Laplacian score (27), a widely used feature selection method, was performed to identify the most representative features from the high-dimensional datasets, i.e., DNAm values of PLS1 genes. Laplacian score was performed as follows:


(2) $$\begin{array}{l} L_{r}=\frac{{\tilde{x}}_{r}^{T}L{\tilde{x}}_{r}}{{\tilde{x}}_{r}^{T}D_{g}{\tilde{x}}_{r}}, \end{array}$$

where *L* is Laplacian Matrix, *D*_*g*_ is the degree matrix of *L*, and ${\tilde{x}}_{r}^{}$ is the center feature of the *r*^th^ original DNAm feature. The DNAm data has *N* features, and the Laplacian score for the methylation level of the *r*^th^ gene is *L*_*r*_.

The CCA mode is aimed at using *a*_*i*_ and *b*_*i*_ to maximize the Pearson correlation between *U*_*i*_ and *V*_*i*_, and then the most important DNAm features associated with the clinical behavioral scores can be selected according to the significance of CCA mode.

Here, we utilized CCA analysis to compute the multivariate correlations between patients' baseline DNAm of PLS1 genes and baseline clinical behavioral scores (PANSS_P, _N, _G), as well as between longitudinal alterations of patients' DNAm of PLS1 genes (baseline DNAm_0W–DNAm_8W) and patients' reduction rate of PANSS_P, PANSS_N, and PANSS_G. During performing CCA, the DNAm values of PLS1 genes and symptomatic scores were standardized with z-score transformation.

Taking the longitudinal data as an example, the CCA analysis started with the construction of epigenetic and symptomatic matrices, *X*_*n*×*s*_ and *Y*_*n*×*t*_ (*n* = 38, *s* = number of representative features of longitudinal alterations of patients' DNAm of PLS1 genes in the Laplacian dimension reduction analysis, *t* = 3 i.e., PANSS_P, _N, and _G). For each CCA mode, we utilized the permutation test (10,000 times) to test the significance of canonical correlations (26). A CCA mode would be valid only if the permutation test (10,000 times) reached significance (*P*_*FWE*_ < 0.05). If at least one significant CCA mode was identified for the PANSS symptoms, the linear associations between columns of the CCA clinical symptom variate (or estimated clinical projection matrix) with each of the original DNAm variables were estimated using univariate variate-to-variable correlations, i.e., Pearson's correlations. All *P*-values in the Pearson's correlation analyses were explicitly corrected by false discovery rate (FDR). Code about the CCA analysis is available at the previously published work of other teams (28, 29).

We then further analyzed whether there are overlapping genes of the first significant CCA mode (CCA1) genes and the dopamine-related genes. We used an online tool *PathDIP* (http://ophid.utoronto.ca/pathDIP) (30) to search dopamine-related gene lists, with the input pathway terms “dopamine,” “dopamine-receptor-mediated,” “dopamine-receptors,” and “dopaminergic.”

## Results

### Demographics and clinical behaviors

We found no significant between-group differences in the demographic characteristics, see Supplementary Table S1. Patients had significant clinical improvement in positive and general psychopathology symptoms (*Ps* < 0.001; Supplementary Table S2), while their alterations in negative symptom dimension showed marginal significance (*P* = 0.060; Supplementary Table S2).

### Between-group comparisons of CT

Compared with healthy volunteers, patients_0W had significant increases of CT in the R_pericalcarine (Supplementary Table S3, *P* = 0.006, FDR corrected), while marginally significant increases in the L_entorhinal, R_entorhinal, and L_pericalcarine cortices (Supplementary Table S3, *P* = 0.0892, 0.0763, and 0.0794 respectively, FDR corrected).

Significant cortical thinning (Supplementary Table S3, *Ps* < 0.05, FDR corrected) were observed in bilateral caudalmiddlefrontal, inferiortemporal, lateralorbitofrontal, parsopercularis, parsorbitalis, parstriangularis, rostralmiddlefrontal, superiorfrontal, superiorparietal (R_superiorparietal showed marginal significance), superiortemporal, supramarginal, as well as L_caudalanteriorcingulate, L_fusiform, L_inferiorparietal, L_medialorbitofrontal, L_middletemporal, L_precentral, R_bankssts, R_ posteriorcingulate, R_precuneus and R_insula in Patients_8W relative to Patients_0W.

### Spatial associations between gene expression and schizophrenia-related CT alterations

We did not identify significant PLS components associated with the case-control *t*-statistic map of the CT measures (*r* = 0.181, *P* > 0.05).

### Spatial associations between longitudinal CT alterations and gene expression

**Figure 2:**
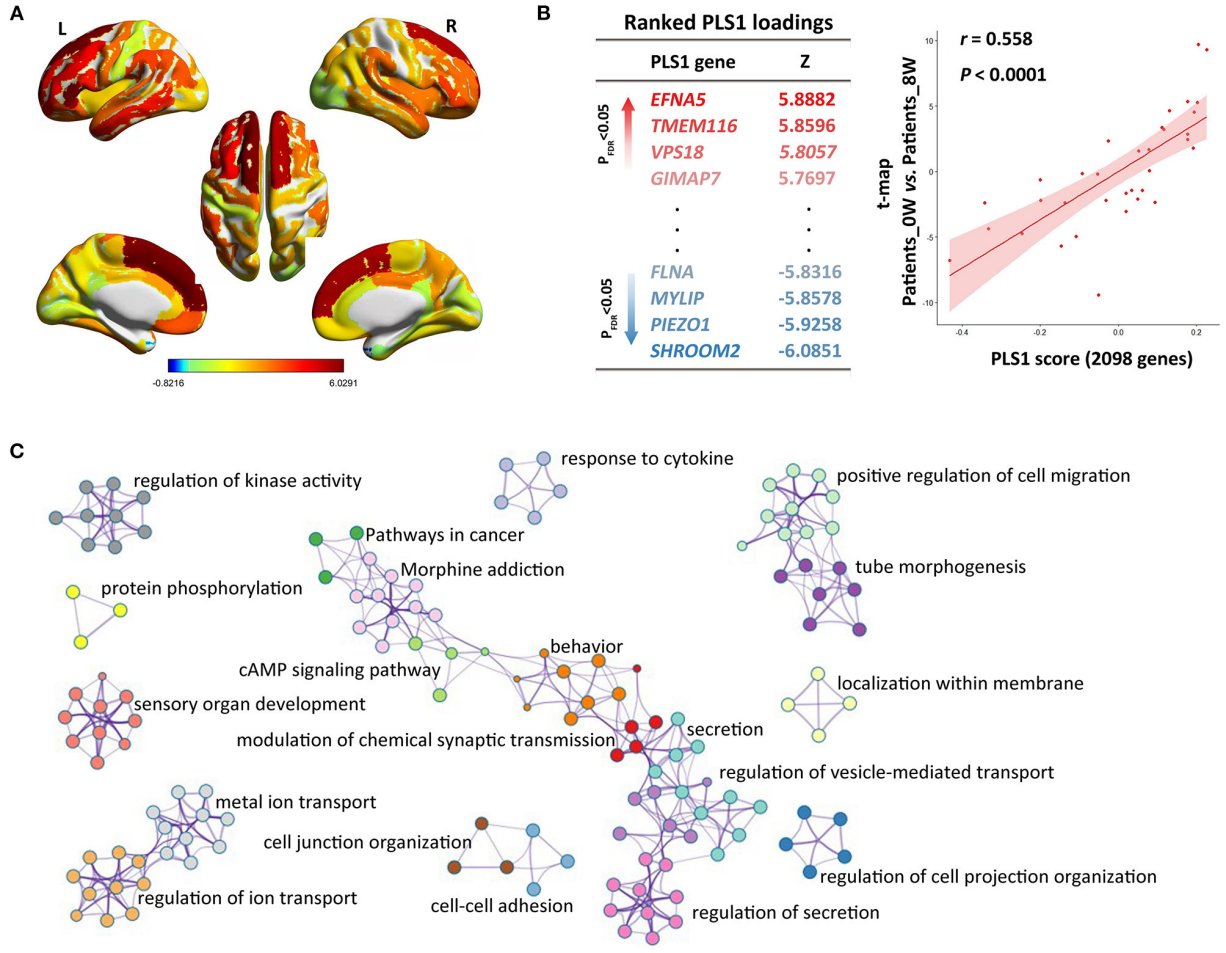
Imaging-transcriptomic analysis treatment-related biomarkers. **(A)** T-statistic map was constructed for the paired comparison of cortical thickness between Patients_0W and Patients_8W; **(B)** Partial least squares (PLS) regression analysis showed that expression levels of 2098 genes in the PLS1 were spatially associated with the above brain image t-map; **(C)** We used Metascape to align the GO biological processes as well as KEGG pathways for the PLS1 genes.

We constructed the t-statistic map representing the longitudinal variations of CT after treatment in the patient group (Figure 2A). As shown in Figure 2B, we detected significant PLS components associated with that t-statistic map (r = 0.558, P < 0.0001), and the PLS1 component had 2,098 genes with normalized PLS1 weights |Z| > 3, including 927 genes with Z > 3, and 1,171 genes with Z < −3 (all P < 0.001 with FDR correction). The PLS1 explained 55.8% of the variance in the longitudinal differences of CT after treatment. We then used Metascape to align the enriched pathways and biological processes for the PLS1 genes. The PLS1 genes were significantly enriched in some neurobiological processes such as modulation of chemical synaptic transmission (with the highest Metascape value), protein phosphorylation and sensory organ development (Figure 2C). They were also enriched in other biological processes such as regulation of cell projection organization, tube morphogenesis, behavior, ion transport, regulation of secretion, regulation of kinase activity, localization within membrane, response to cytokine, et al. (Ps < 0.01 with FDR correction, detailed see Supplementary Table S4). As to the KEGG pathways, the PLS1 genes were significantly enriched in cancer, cAMP signaling and morphine addiction pathways (Ps < 0.01 with FDR correction).

We did not detect significant PLS components associated with the case-control t-statistic map of the CT measures (P > 0.05).

### CCA analysis: Correlation between DNAm of PLS1 genes and clinical symptoms

Based on the above detected 2,098 PLS1 genes, we further analyzed which genes' methylation levels were related to patients' clinical symptoms. Combining Laplacian score and CCA analysis, we linked DNAm of 33 representative genes (CCA1 loading genes) with three dimensions of PANSS symptoms, i.e., PANSS_P, _N, and _G. For the multivariate correlations between patients' baseline DNAm of PLS1 genes and baseline clinical behaviors scores, we detected no significant CCA modes (P_FWE_ > 0.05).

**Figure 3:**
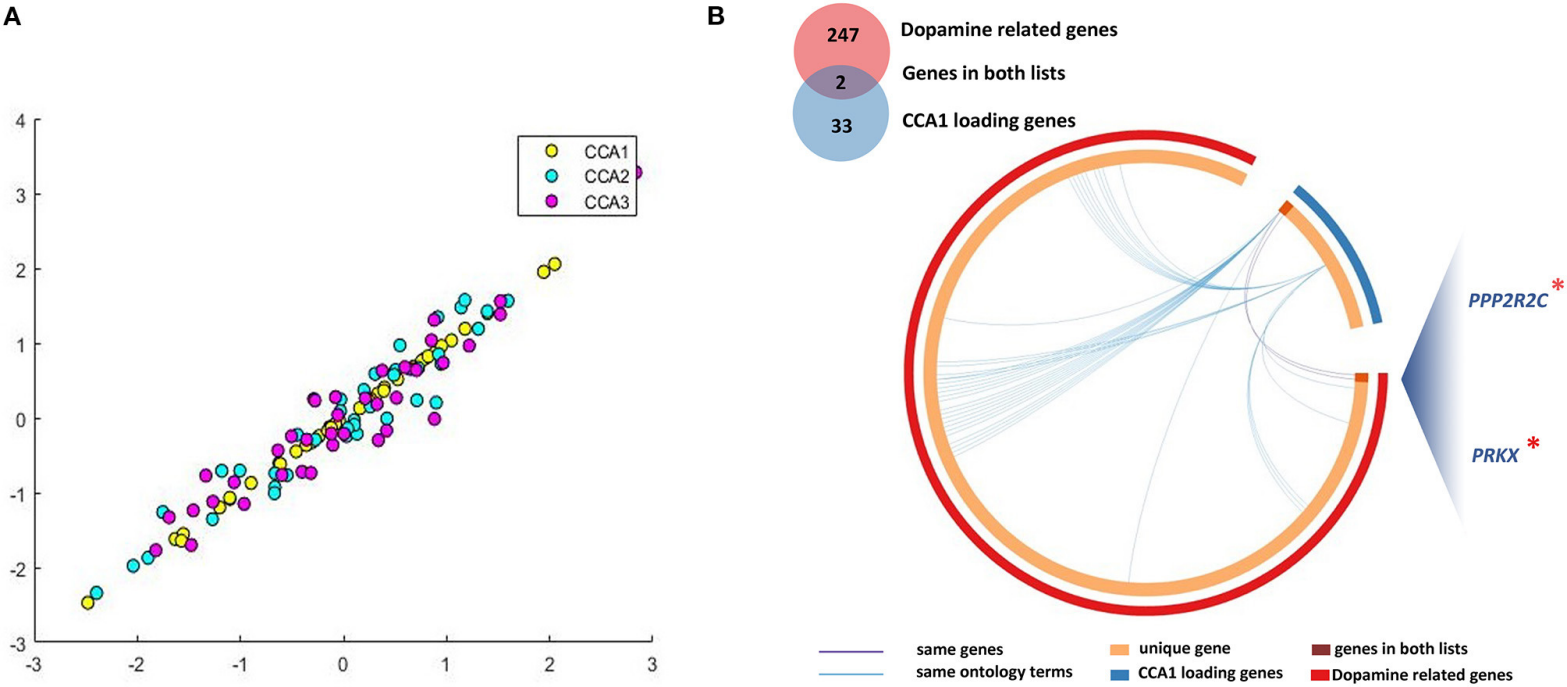
Multivariate correlations between DNAm of CCA loading genes and clinical symptoms. **(A)** We identified significant CCA1 loading genes (33 representative features) associated with patients' longitudinal alterations of clinical symptoms; **(B)** There are two overlapping genes of 33 CCA loading genes and the 247 dopamine-related genes. CCA, canonical correlation analysis. *, important genes overlapping between dopamine related genes and CCA1 loading genes.

For the analysis between longitudinal alterations of patients' DNAm of PLS1 genes (DNAm_0W–DNAm_8W) and patients' reduction rate of PANSS_P, PANSS_N, and PANSS_G, the first CCA mode (CCA1) was significantly correlated (P_FWE_ = 0.0174, Figure 3A). To quantify which DNAm variables were significantly involved in the first CCA mode, we then analyzed univariate variable-to-variate Pearson's correlations between the DNAm variables and the corresponding PANSS variate, and found 33 significant genes with P_FDR_ < 0.05 (see Table 1). The corresponding PLS Z-values of the 33 genes computed in the above imaging-transcriptomic spatial association analysis were also shown in Table 1. There are two overlapping genes of 33 CCA loading genes and the 247 dopamine-related genes (obtained from the PathDIP), i.e., PPP2R2C and PRKX (Figure 3B).

**Table 1:** Significant genes in the CCA analysis between longitudinal DNAm of PLS1 genes and patients' reduction rate of clinical symptoms.

**Genes**	***P*** **_FWE_ of mode 1**	**PLS *Z*-value**	**Genes**	***P*** **_FWE_ of mode 1**	**PLS *Z*-value**	**Genes**	***P*** **_FWE_ of mode 1**	**PLS *Z*-value**
ITGB3BP	0.0070	5.076999	SPTLC2	0.0073	3.338194	ZDHHC12	0.0077	−3.63217
LRRFIP2	0.0069	4.873722	TRAPPC9	0.0069	3.226657	PRKX	0.0074	−3.69343
STAG2	0.0072	4.694063	USP6NL	0.0070	3.056709	KRT19	0.0047	−3.75481
CALD1	0.0077	4.679627	PYROXD1	0.0069	−3.12342	NOSTRIN	0.0079	−3.83761
SLC20A1	0.0076	4.612949	ZNF667	0.0077	−3.14007	INPP5J	0.0070	−4.0137
CC2D1A	0.0057	4.377086	PELI1	0.0071	−3.21708	CDH12	0.0036	−4.08813
DNAJC12	0.0070	4.225474	ZNF772	0.0075	−3.22121	TNNC2	0.0057	−4.22949
NPY	0.0029	3.777707	COL11A1	0.0052	−3.39214	IER2	0.0073	−4.23121
PARD6G	0.0067	3.753459	DCHS1	0.0052	−3.44326	SNAPC2	0.0055	−4.25052
SLN	0.0037	3.563281	PPP2R2C	0.0070	−3.51934	ARHGAP19	0.0070	−4.79779
HYI	0.0071	3.557632	ATP6V0A2	0.0036	−3.53321	B3GNT5	0.0071	−5.47048

PLS, partial least squares.

## Discussion

This study firstly investigates multi-omics biomarkers associated with antipsychotic treatment in the first episode schizophrenia using multiple measures linking CT, public transcriptomic signatures and peripheral epigenetic modifications. As hypothesized, alterations of CT and clinical symptoms after antipsychotic treatment may be transcriptomically and epigenetically underlied. Phenotypic variations of CT related to treatment were spatially associated with expression of genes (PLS1 genes) primarily enriched in neurobiological processes, as well as dopaminergic related and cancer pathways. Moreover, the longitudinal alterations of DNAm for the 33 of PLS1 genes demonstrated significant associations with patients' reduction rate of clinical symptoms.

In this study, following 8 weeks of risperidone treatment, brain cortex showed significant thinning primarily in the frontal and temporal lobes, but also in parts of parietal regions. Similarly, one most recent randomized, double-blind, placebo-controlled study found that patients with major depressive disorder exposed to olanzapine had cortical thinning throughout all lobes across a 36-week period compared with those taking a placebo (31). Cross-sectional studies of patients with schizophrenia undergoing chronic (over 5 years) or current treatment suggest excessive thinning in widespread brain regions such as the middle temporal, prefrontal, occipital and parietal regions, and the excessive cortical thinning, progressing across the entire disease course, appears associated with medication intake (3–7). Contrary to our findings, however, a prospective longitudinal study with a sample of 34 unmedicated patients with schizophrenia demonstrated significant increase of CT after shorter term (6 weeks) of acute-phase treatment with risperidone, and the greater increase were correlated with better clinical response (32). Future studies are needed to investigate the potential different effects of short-term 6- and 8-week atypical treatment on human cerebral CT in first-episode patients.

Interestingly, patients' longitudinal variances of CT after treatment showed significant spatial correlations with gene expression of cortical areas derived from AHBA (PLS1 genes). The detected genes were enriched in multiple biological processes including some neurobiological processes, e.g., modulation of chemical synaptic transmission and protein phosphorylation. Consistently, the action of antipsychotic drugs mainly depends on chemical synaptic transmission of dopaminergic neurons (33); protein phosphorylation alterations have been previously suggested to play a vital role in mediating the treatment effects (34). Together with these discoveries, it is plausible to propose that the decreased CT found in this study could be due to atypical induced synaptic remodeling and neural plasticity documented in the frontal, temporal and parietal cortices. Besides, we also found that genes involving the sensory organ development may underly the treatment-related phenotypic variations in CT. Abnormal processing of auditory (35), visional (36), gustative (37), olfactory (38), and tactile (39) stimuli has been previously suggested to be potential markers of mental disorders including schizophrenia. Our current finding would expand the pipeline of ideas for developing new targets of antipsychotic drug action.

As to the KEGG pathways, the PLS1 genes were significantly enriched in cAMP signaling and morphine addiction and cancer pathways. Previous studies have revealed that proteins downstream of cAMP-related pathway, the main messenger for signaling through the dopamine receptors (40), are altered after acute antipsychotic therapy (41); dopamine receptors, the main targets of antipsychotic drugs, could modulate limbic morphine-induced plasticity (42). It would therefore be reasonable to speculate that longitudinal CT alterations after 8-week risperidone treatment may be driven by transcriptional status of dopaminergic signaling associated pathways. Interestingly, the transcriptional state for the cancer pathway may also underlie the longitudinal phenotypic variances in cortical morphology. Recently, antipsychotic agents are extensively being tested for efficacy in patients with various cancers. For example, chlorpromazine may harbor effects on treating human oral cancer (43). In addition, due to their proven ability to cross the blood–brain barrier, antipsychotic medications were also found to have effect on malignant brain tumors (44). These studies reveal the strategies of using the existing antipsychotic agents for cancer, known as “drug repositioning” or “drug repurposing,” and simultaneously imply that the cancer pathway may serve as a possible target for new antipsychotic drug discovery.

DNAm was found to underlie the known heterogeneity of treatment response. Specifically, for the analysis between longitudinal alterations of patients' DNAm of PLS1 genes and patients' reduction rate of PANSS_P, PANSS_N, and PANSS_G, the first CCA mode was significantly correlated. To quantify which DNAm variables were significantly involved in the first CCA mode, we then analyzed univariate variable-to-variate Pearson's correlations between the DNAm variables and the corresponding PANSS variate, and found 33 significant genes, which overlap with two of the dopamine-related genes, i.e., *PPP2R2C* and *PRKX*. Evidence from both transcriptional and epigenetic omics simultaneously demonstrated the importance of dopamine signals in antipsychotic treatment and treatment response.

Our findings must be explained in light of some limitations. First, the sample size is small due to the challenging requirement of drug-naïve patients with first-episode schizophrenia given antipsychotic (risperidone) monotherapy. The current findings are still needed to be replicated in other cohorts with large sample size. Second, we did not calculate the gene expression data (from the AHBA) of the right hemisphere, because not all donors have brain tissues of the right hemisphere. Thus, the association between gene expression and treatment-related alterations in CT does not represent the condition of bilateral cortical regions. Third, the methylation levels were derived from peripheral blood rather than brain tissues of the cortical regions, although DNAm values were significantly correlated between brain and blood (13).

## Conclusions

This study firstly revealed that changes of CT and clinical behaviors after treatment may be transcriptionally and epigenetically underlied. Phenotypic variations of cortical thickness related to treatment were spatially associated with expression of genes primarily enriched in neurobiological processes, as well as dopaminergic related and cancer pathways. We define a “three-step” roadmap which represents a vital step toward the exploration of treatment- and treatment response-related biomarkers on the basis of multiple omics rather than a single omics type as a strategy for advancing precise care.

## Data availability statement

The datasets presented in this study can be found in online repositories. The names of the repository/repositories and accession number(s) can be found in the article/Supplementary material.

## Ethics statement

The studies involving human participants were reviewed and approved by the Ethics Committee of Henan Mental Hospital and The Second Xiangya Hospital of Central South University. The patients/participants provided their written informed consent to participate in this study.

## Author contributions

Conceptualization: XZ, MH, GW, SW, QT, and JZ. Participants recruitment: GW, ZN, SM, LK, and NZ. Image analysis: MH and JZ. Multi-omics analysis: MH and QT. Writing—original draft preparation: XZ. Writing—review and editing: XZ, MH, SW, QT, and JZ. Supervision and project administration: MH, SW, QT, and JZ. All authors contributed to the final version of the paper.
