# Supplementary material for: Longitudinal multi-omics alterations response to 8-week risperidone monotherapy: Evidence linking cortical thickness, transcriptomics and epigenetics
Source: Front Psychiatry. 2023 Mar 2;14:1127353. doi: 10.3389/fpsyt.2023.1127353 (PMC10018025; doi:10.3389/fpsyt.2023.1127353)
Supplement: Supplementary file 6 [file Data_Sheet_1.docx]

%% data load

% PLS code

Nor_data = readtable('basic_data.xlsx','PreserveVariableNames',true,'Sheet', 1);

Com_data = readtable('basic_data.xlsx','PreserveVariableNames',true,'Sheet', 2);

% regression t_map 1

t_map_matrix1 = zeros(34,3);

for i = 1:34

X_input = table2array(Nor_data(:,36:38));

Y_output = table2array(Nor_data(:,i+1));

[~,~,r] = regress(Y_output,X_input);

Nor_in = r(1:38);

Cure_in = r(39:end);

[h,p,ci,stats] = ttest2(Nor_in,Cure_in,'Alpha',0.05);

t_value = stats.tstat;

t_map_matrix1(i,1) = h;

t_map_matrix1(i,2) = p;

t_map_matrix1(i,3) = t_value;

end

% regression t_map 2

t_map_matrix2 = zeros(34,3);

for i = 1:34

X_input = table2array(Com_data(:,36:38));

Y_output = table2array(Com_data(:,i+1));

[~,~,r] = regress(Y_output,X_input);

Nor_in = r(1:38);

Cure_in = r(39:end);

[h,p,ci,stats] = ttest(Nor_in,Cure_in,'Alpha',0.05);

t_value = stats.tstat;

t_map_matrix2(i,1) = h;

t_map_matrix2(i,2) = p;

t_map_matrix2(i,3) = t_value;

end

%% data load

nq_label = readtable('34¸öÄÔÇøÃû×Ö.xlsx','Sheet', 1);

gen_label = readtable('10027¸ö»ùÒòÃû³Æ.xlsx','PreserveVariableNames',true,'Sheet', 1);

gen_data = readtable('34ÄÔÇø³Ë10027»ùÒò.xlsx','Sheet', 1);

gen_data = table2array(gen_data);

%% Gene PLS % t-map 1

% Z-score

X0 = gen_data;

Y0 = t_map_matrix2(:,3); % t_map_matrix1 or t_map_matrix2

X = zscore(X0);

Y = zscore(Y0);

% [XL,YL] = plsregress(X,Y,1);

[XL,YL,XS,YS,BETA,PCTVAR,MSE,stats] = plsregress(X,Y,1);

disp(PCTVAR(1));

[r_val,p_val] = corr(XS,Y,'Type','Pearson');

% figure

% plot(XS,Y,'r.')

% xlabel('XS scores for PLS component 1','FontSize',14);

% ylabel('t map','FontSize',14);

% grid on

% [r_vals,p_vals] = corr(XS,YS,'Type','Pearson');

r_val_gene = zeros(10027,1);

p_val_gene = zeros(10027,1);

p_zval_gene = zeros(10027,1);

% [h,p,ci,zval] = ztest(X0(:,1),0,1,0.05);

for i = 1:10027

[r_val_gene(i),p_val_gene(i)] = corr(XS,X(:,i),'Type','Pearson');

[~,p_zval_gene(i)] = ztest(X0(:,i),0,1);

end

% FDR adjust

p_fdr = mafdr(p_zval_gene);

length(find (p_fdr<=0.05))

% index_pls1 = find(abs(XL)>=3);

% index_pls2 = find(abs(XL)>=4);

% index_pls3 = find(abs(XL)>=5);

% p_fdr = mafdr(p_val_gene);

% length(find (p_val_gene<=0.05))

%

% index_pls1 = find(abs(XL)>=3);

% index_pls2 = find(abs(XL)>=4);

% index_pls3 = find(abs(XL)>=5);

%% permutationTest

% r_val_set = zeros(1000,1);

% for i = 1:1000

% Y1 = zscore(Y0(randperm(34)));

% [~,~,XS1,~,~,~] = plsregress(X,Y1,1);

% [r_val1,~] = corr(XS1,Y,'Type','Pearson');

% r_val_set(i) = r_val1;

% end

%

% r_val_sort = sort(abs(r_val_set),'descend');

% num = length(find(r_val_sort>=abs(r_val)));

% disp(num);

% figure

% histogram(r_val_set)

% xlabel('r value');

% ylabel('Count')

% legend(sprintf('r value = %.2f,\np = %f\n num = 1', r_val, p_val));

%% permutation Test S

rep=1000;

for dim=1:8

[XL,YL,XS,YS,BETA,PCTVAR,MSE,stats]=plsregress(X,Y,dim);

temp=cumsum(100*PCTVAR(2,1:dim));

Rsquared = temp(dim);

for j=1:rep

%j

order=randperm(size(Y,1));

Yp=Y(order,:);

[XL,YL,XS,YS,BETA,PCTVAR,MSE,stats]=plsregress(X,Yp,dim);

temp=cumsum(100*PCTVAR(2,1:dim));

Rsq(j) = temp(dim);

end

dim

R(dim)=Rsquared

p(dim)=length(find(Rsq>=Rsquared))/rep

end

figure

plot(1:dim, p,'ok','MarkerSize',8,'MarkerFaceColor','r');

xlabel('Number of PLS components','FontSize',14);

ylabel('p-value','FontSize',14);

%%

% [r_vals,p_vals] = corr(XS,YS,'Type','Pearson');

% r_vals_set = zeros(1000,1);

% for i = 1:1000

% Y1 = zscore(Y0(randperm(34)));

% [~,~,XS1,YS1,~,~] = plsregress(X,Y1,1);

% [r_val1,~] = corr(XS1,YS1,'Type','Pearson');

% r_vals_set(i) = r_val1;

% end

%

% r_vals_sort = sort(r_vals_set,'descend');

% num = length(find(r_vals_sort>=abs(r_vals)));

% disp(num);

% figure

% histogram(r_vals_set)

% xlabel('r value');

% ylabel('Count')

% legend(sprintf('r value = %.2f,\np = %f\n num = 342', r_vals, p_vals));

%num 11/1000 1

%num 0/1000 2

%num 0/1000 3

%% bootstraps

%number of bootstrap iterations:

bootnum=1000;

% Do PLS in 2 dimensions (with 2 components):

dim=2;

[XL,YL,XS,YS,BETA,PCTVAR,MSE,stats]=plsregress(X,Y,dim);

%store regions' IDs and weights in descending order of weight for both components:

[R1,p1]=corr([XS(:,1),XS(:,2)],Y);

%align PLS components with desired direction for interpretability

if R1(1,1)<0 %this is specific to the data shape we were using - will need ammending

stats.W(:,1)=-1*stats.W(:,1);

XS(:,1)=-1*XS(:,1);

end

if R1(2,1)<0 %this is specific to the data shape we were using - will need ammending

stats.W(:,2)=-1*stats.W(:,2);

XS(:,2)=-1*XS(:,2);

end

[PLS1w,x1] = sort(stats.W(:,1),'descend');

% PLS1ids=genes(x1);

% geneindex1=geneindex(x1);

[PLS2w,x2] = sort(stats.W(:,2),'descend');

% PLS2ids=genes(x2);

% geneindex2=geneindex(x2);

%define variables for storing the (ordered) weights from all bootstrap runs

PLS1weights=[];

PLS2weights=[];

%start bootstrap

for i=1:bootnum

% i

myresample = randsample(size(X,1),size(X,1),1);

res(i,:)=myresample; %store resampling out of interest

Xr=X(myresample,:); % define X for resampled subjects

Yr=Y(myresample,:); % define X for resampled subjects

[XL,YL,XS,YS,BETA,PCTVAR,MSE,stats]=plsregress(Xr,Yr,dim); %perform PLS for resampled data

temp=stats.W(:,1);%extract PLS1 weights

newW=temp(x1); %order the newly obtained weights the same way as initial PLS

if corr(PLS1w,newW)<0 % the sign of PLS components is arbitrary - make sure this aligns between runs

newW=-1*newW;

end

PLS1weights=[PLS1weights,newW];%store (ordered) weights from this bootstrap run

temp=stats.W(:,2);%extract PLS2 weights

newW=temp(x2); %order the newly obtained weights the same way as initial PLS

if corr(PLS2w,newW)<0 % the sign of PLS components is arbitrary - make sure this aligns between runs

newW=-1*newW;

end

PLS2weights=[PLS2weights,newW]; %store (ordered) weights from this bootstrap run

end

%get standard deviation of weights from bootstrap runs

PLS1sw=std(PLS1weights');

PLS2sw=std(PLS2weights');

%get bootstrap weights

temp1=PLS1w./PLS1sw';

temp2=PLS2w./PLS2sw';

%order bootstrap weights (Z) and names of regions

[Z1,ind1]=sort(temp1,'descend');

% PLS1=PLS1ids(ind1);

% geneindex1=geneindex1(ind1);

[Z2,ind2]=sort(temp2,'descend');

% PLS2=PLS2ids(ind2);

% geneindex2=geneindex2(ind2);

gene_ind = ind1(x1);

% Z1S = Z1(ind1);

indd = find(abs(Z1)>=3);

length(find(abs(Z1)>=3))

z_value = Z1;

% save result

save result20220126_test1_2.mat

%% ## ##

%% 20220126

% one sample Z test

temp_data = table(table2array(gen_label(gene_ind,:)),z_value,p_fdr(gene_ind),'VariableNames',{'gen_label','z_value','p_fdr'});

temp_data1 = temp_data(indd,:);

indext = find(abs(table2array(temp_data1(:,3)))<=0.05);

length(find(abs(table2array(temp_data1(:,3)))<=0.05))

temp_data2 = temp_data1(indext,:);

writetable(temp_data2,'temp_test1.xlsx')

%% stop here

%%

% temp_data = readtable('temp.xlsx','PreserveVariableNames',true,'Sheet', 1);

temp_data = table(table2array(gen_label(gene_ind,:)),z_value,'VariableNames',{'gen_label','z_value'});

temp_data1 = temp_data(indd,:);

writetable(temp_data1,'temp_test1.xlsx')

%% ## ##

%% old version

% one sample Z test

temp_data = table(table2array(gen_label(gene_ind,:)),z_value,r_val_gene(gene_ind),p_fdr(gene_ind),'VariableNames',{'gen_label','z_value','r_val','p_fdr'});

temp_data1 = temp_data(indd,:);

indext = find(abs(table2array(temp_data1(:,4)))<=0.05);

length(find(abs(table2array(temp_data1(:,4)))<=0.05))

temp_data2 = temp_data1(indext,:);

writetable(temp_data2,'temp_test1.xlsx')

%%

%% bootstrap

BETA_Set = zeros(500,10027);

for i = 1:500

list = zeros(34,1);

for j = 1:34

list(j) = randperm(length(Y0),1);

end

XB = X0(list,:);

YB = Y0(list,:);

X = zscore(XB);

Y = zscore(YB);

% [XL,YL] = plsregress(X,Y,1);

[~,~,~,~,BETAS,~] = plsregress(X,Y,1);

BETA_Set(i,:) = BETAS(2,:)';

end

BETA_Std = std(BETA_Set);

BETA_SE = BETA_Std./sqrt(500);

Z_Value_Set = zeros(10027,1);

for i = 1:10027

Z_Value_Set(i) = BETA(i+1) / BETA_SE(i);

end

figure

histogram(Z_Value_Set)

xlabel('Z Value');

ylabel('Count')

% legend(sprintf('abs(Z Value)>=5\nnum = 348'));

% length(find(abs(Z_Value_Set)>=5))

Z_Value_Set_Zscore = zscore(Z_Value_Set);

figure

histogram(Z_Value_Set_Zscore)

xlabel('Z Value for Zscore');

ylabel('Count')

legend(sprintf('abs(Z Value Zscore)>=1.96\nnum = 348'));

index1 = find(abs(Z_Value_Set_Zscore)>=1.96);

index2 = find(abs(Z_Value_Set_Zscore)>=3);

length(find(abs(Z_Value_Set_Zscore)>=3))

length(find(abs(Z_Value_Set_Zscore)>=1.96))

% save result20220121.mat

indexZ1 = find(abs(Z_Value_Set)>=3);

indexZ2 = find(abs(Z_Value_Set)>=4);

indexZ3 = find(abs(Z_Value_Set)>=5);

temp_data = readtable('temp.xlsx','PreserveVariableNames',true,'Sheet', 1);

temp_data1 = temp_data(index1,:);

writetable(temp_data1,'temp2.xlsx')

indext = find(abs(table2array(temp_data1(:,3)))<=0.05);

temp_data2 = temp_data1(indext,:);

writetable(temp_data2,'temp3.xlsx')

length(find(abs(table2array(temp_data1(:,3)))<=0.05))

%

% [bootstat,bootsam] = bootstrp(500,@(c) std(c) , X);

%

% function [] = boostrapv()

%

%

% end

% [r_val,p_val] = corr(YS,X,'Type','Pearson');

% res = find(p_val<=0.05);

% datas = r_val(res);

% hist(r_val)

% [XL,YL] = plsregress(X,Y,ncomp)

% %% permutationTest

% % change the order and test the result

% Covariance = diag(cov(X0));

% PLS1 = XL(:,1);

% permutationTest(PLS1, Covariance, 500, ...

% 'plotresult', 1, 'showprogress', 250)

%

% permutationTest(PLS1, Covariance, 1000, ...

% 'plotresult', 1, 'showprogress', 250)

% % permutationTest(sample1, sample2, permutations [, varargin])

%% bootstrap

[bootstat,bootsam] = bootstrp(500,@(c) std(c) , X);

% [bootstat,bootsam] = bootstrp(500,@(c) std(c) , X);

% [bootstat,bootsam] = bootstrp(500,@(c) [mean(c)] , X);

% bootstat = bootstrp(nboot,bootfun,d)
