# Supplementary material for: Longitudinal multi-omics alterations response to 8-week risperidone monotherapy: Evidence linking cortical thickness, transcriptomics and epigenetics
Source: Front Psychiatry. 2023 Mar 2;14:1127353. doi: 10.3389/fpsyt.2023.1127353 (PMC10018025; doi:10.3389/fpsyt.2023.1127353)
Supplement: Supplementary file 7 [file Data_Sheet_2.docx]

Longitudinal multi-omics alterations response to 8-week risperidone monotherapy: evidence linking cortical thickness, transcriptomics and epigenetics

Xiaofen Zong ^a^, Gaohua Wang ^a^, Zhaowen Nie ^a^, Simeng Ma ^a^, Lijun Kang ^a^, Nan Zhang ^a^, Shenhong Weng ^a,^*, Qing Tan ^b, c,^*, Junjie Zheng ^d, e,^* and Maolin Hu ^a,f,g,^*

^a^ Department of Psychiatry, Renmin Hospital of Wuhan University, Wuhan, Hubei, China

^b^ School of Mathematics and Statistics, Wuhan University, Wuhan, Hubei, China

^c^ Hubei Key Laboratory of Computational Science, Wuhan University, Wuhan, Hubei, China

^d^ The Early Intervention Unit, Department of Psychiatry, Affiliated Nanjing Brain Hospital, Nanjing Medical University, Nanjing, Jiangsu, China

^e^ The Functional Brain Imaging Institute, Nanjing Medical University, Nanjing, Jiangsu, China

^f^ Department of Psychiatry, the Second Xiangya Hospital of Central South University, Changsha, Hunan, China

^g^ Department of Psychiatry, Henan Mental Hospital, the Second Affiliated Hospital of Xinxiang Medical University, Xinxiang, Henan, China

***** Corresponding author at: Department of Psychiatry, Renmin Hospital of Wuhan University, 238 Jiefang Road, Wuhan, Hubei, China.

E-mail address: [humaolin@whu.edu.cn](mailto:humaolin@whu.edu.cn) (M. Hu); wengshenhong@whu.edu.cn (S. Weng);

***** Corresponding author at: School of Mathematics and Statistics, Wuhan University, Wuhan, Hubei, China; Hubei Key Laboratory of Computational Science, Wuhan University, Wuhan, Hubei, China.

E-mail address: [qtan@whu.edu.cn](mailto:qtan@whu.edu.cn) (Q. Tan);

***** Corresponding author at: The Early Intervention Unit, Department of Psychiatry, Affiliated Nanjing Brain Hospital, Nanjing Medical University, Nanjing, Jiangsu, China; The Functional Brain Imaging Institute, Nanjing Medical University, Nanjing, Jiangsu, China.

E-mail address: [zjj5270@163.com](mailto:zjj5270@163.com) (J. Zheng)

**Supplementary Material and Methods**

**T1 imaging acquisition**

We obtained T1-weighted images (sagittal view) with standard 16-channel head coil. The scanning sequence was spoiled gradient echo pulse: time of echo, 2.52 ms; time of repetition, 1900 ms; field of view, 250 × 250 mm^2^; flip angle, 9°; gap, 0 mm; thickness, 1.0 mm; slices, 176.

**Image analysis**

Cortical thickness estimation was automatically conducted with the FreeSurfer software package (v5.3.0). Image preprocessing includes: 1) correcting head motion; 2) stripping skull; 3) Talairach transformation; and 4) segmentation. We conducted the analysis process with no manual intervention. More details about technical processing were also shown in the online pipeline (<http://surfer.nmr.mgh.harvard.edu/>).

**Preprocessing of AHBA data**

The brain gene expression data were acquired from the post-mortem brain tissues of six donors of AHBA, consisting of three European-ancestry male (H0351.1009, 57-year-old; H0351.1012, 31-year-old; and H0351.1016, 55-year-old), one Hispanic female (H0351.1015, 49-year-old), and two African American males (H0351.2001, 24-year-old; and H0351.2002, 39-year-old). The AHBA includes microarray data in 3702 samples (brain tissues) which are spatially distincted and taken from 6 neurotypical adult brains. The samples (brain tissues) consist of subcortical, cortical, cerebellar and brainstem areas in each brain, and quantify the transcriptional levels of more than 20,000 genes. More details about the public brain expression data are also available at http://www.brain-map.org.

**PLS Regression Analysis**

Each AHBA brain tissue sample relate to a numeric structure ID, structure label (cerebellum, cortex or brainstem) and name in addition to the voxel-level MRI coordinates in native neuroimaging space and stereotaxic MNI coordinates, which can be utilized to match brain tissues to the neuroimaging t-maps of our current study.

After 5-step preprocessing, the 6 AHBA transcriptome data were processed into 34 (brain regions)×10027 (probes) data set. In the PLS analysis, expression data of the 10027 probes for the 34 brain regions were predictor variables, the t-statistic map (for 34 brain regions) was set as response variables. In the PLS analysis, all the transcriptomes (34×10027) were put into the PLS model. In the PLS analysis, expression data of the 10027 probes for the 34 brain regions were predictor variables, the t-statistic map (for 34 brain regions) was set as response variables.

**DNA Extraction**

The DNA of peripheral blood cells in all the 114 samples (38 healthy controls, 38 Patients_0W, and 38 Patients_8W) was isolated by using DNA Blood Mini Kit (Qiagen). The ratio of A260/A280, calculated with the NanoDrop (Thermo Scientific), was utilized to assess DNA purity. We used Agarose gel electrophoresis to detect the DNA quality.

**Bisulfite Conversion**

We used sodium bisulfite to treat the genomic DNA of all the 114 samples. Then, the sodium bisulfite-treated DNA was quantified with the EZ DNAm kit (Zymo Research; Irvine, CA).

**Illumina 450K Genechip**

Following the manufacturer's recommended instructions, we measured the DNAm status with the Infinium® Human Methylation 450K BeadChip (Illumina Inc, San Diego, CA). We randomly distributed all the DNA smaples of the 114 participants to different arrays in order to control the positional/batch effects.

**The Quality Control of Genechip Assay**

The quality control (QC) of all the genechips was tested on the GenomeStudio software (v1.9, methylation module). The QC processes consisted of sample-dependent and sample-independent controls. The sample-dependent QC included bisulfite conversion, stringency, and negative and non-polymorphic QC; the sample-independent QC included extension, staining, target removal and hybridization controls.

We also corrected the positional and batch effect using the ComBat empirical Bayesian method ([1](#_ENREF_1)). The reference-based method was performed to assess blood cell type proportions ([2](#_ENREF_2)). We conducted linear regression to regress out the effects of cell type proportion as well as age and sex for each probe. The mean level of all CpG sites in each gene was assessed to represent the DNAm level of this gene. Among the 114 samples, this study utilized the DNAm data of 38 samples of Patients_0W and 38 of Patients_8W.

**Illumina 450K Microarray Processing**

We performed the default parameters on the GenomeStudio software (v1.9, methylation module) to process the Illumina array data. We normalized raw data with the Lumi and BMIQ R packages, which include probe type bias as well as color-bias adjustment. Lumi was conducted for the convertion of the individual CpG sites into methylation levels “β-values” (ranging from 0 to 1). The β-values were calculated as the ratio of the methylated intensity to the sum of unmethylated and methylated signal intensity.

**Supplementary Results**

**Table S1** Demographic information of case and control groups

| **Variable** | **Controls (n=38)** | | **Patients (n=42)** | | **Analysis^a^** | |
| --- | --- | --- | --- | --- | --- | --- |
|  | M | SD | M | SD | t | P |
| Age (years) | 24.76 | 4.56 | 24.86 | 4.80 | 0.09 | 0.929 |
| Education | 11.05 | 2.91 | 10.48 | 2.84 | 0.90 | 0.373 |
|  | Yes | No | Yes | No | *χ*^2^ | P |
| Gender (male) | 25 | 13 | 27 | 15 | 0.02 | 0.888 |
| Handedness (right) | 38 | 0 | 42 | 0 |  |  |

^a^ two-sample t test, patients *vs.* controls.

**Table S2** Patients’ clinical symptom alterations after 8-week treatment

| **Variables** | **Patients before treatment (n=42)** | | **Patients after treatment (n=38)** | | **Analysis^a^** | |
| --- | --- | --- | --- | --- | --- | --- |
|  | M | SD | M | SD | t | P |
| PANSS-T | 91.90 | 11.23 | 66.32 | 9.91 | 14.78 | <0.001 |
| PANSS-G | 48.14 | 6.46 | 34.16 | 4.84 | 13.47 | <0.001 |
| PANSS-P | 25.60 | 3.75 | 15.39 | 2.98 | 19.15 | <0.001 |
| PANSS-N | 18.17 | 5.21 | 16.76 | 4.44 | 1.94 | 0.060 |

Note: Four patients withdrew the follow-up scans. ^a^patients at baseline (n=38) *vs.* patients at follow-up (n=38), paired-sample t test. Abrreviations: PANSS = Positive and Negative Syndrome Scale; PANSS-P = PANSS positive symptom scores; PANSS-G = PANSS general psychopathological symptom scores; PANSS-N = PANSS negative symptom scores; PANSS-T = PANSS total scores.

**Table S3** Significant between-group differences in cortical thickness

| **Cortical thickness** | **Patients_0W vs. Healthy controls^a^** | | | **Patients_0W vs. Patients_8W^b^** | | |
| --- | --- | --- | --- | --- | --- | --- |
| Schizophrenia-related differences | *t* | *P* | *P_FDR_* | *t* | *P* | *P_FDR_* |
| L_entorhinal | -2.287 | 0.0249 | 0.0892^c^ | NA | NA | NA |
| R_entorhinal | -2.382 | 0.0196 | 0.0763^c^ | NA | NA | NA |
| L_pericalcarine | -2.344 | 0.0216 | 0.0794^c^ | NA | NA | NA |
| R_pericalcarine | -3.614 | 0.0005 | 0.0060 | NA | NA | NA |
| Treatment-related differences | *t* | *P* | *P_FDR_* | *t* | *P* | *P_FDR_* |
| L_caudalmiddlefrontal | NA | NA | NA | 4.519 | 6.16E-05 | 0.0017 |
| R_caudalmiddlefrontal | NA | NA | NA | 3.000 | 4.80E-03 | 0.0261 |
| L_inferiortemporal | NA | NA | NA | 3.869 | 0.0004 | 0.0060 |
| R_inferiortemporal | NA | NA | NA | 2.844 | 0.0072 | 0.0377 |
| L_lateralorbitofrontal | NA | NA | NA | 3.341 | 0.0019 | 0.0153 |
| R_lateralorbitofrontal | NA | NA | NA | 2.797 | 0.0081 | 0.0410 |
| L_parsopercularis | NA | NA | NA | 4.268 | 0.0001 | 0.0026 |
| R_parsopercularis | NA | NA | NA | 3.005 | 0.005 | 0.0261 |
| L_parsorbitalis | NA | NA | NA | 3.702 | 0.0007 | 0.0072 |
| R_ parsorbitalis | NA | NA | NA | 3.795 | 0.0005 | 0.0060 |
| L_parstriangularis | NA | NA | NA | 5.893 | 8.75E-07 | 0.00006 |
| R_parstriangularis | NA | NA | NA | 3.891 | 4.01E-04 | 0.0060 |
| L_rostralmiddlefrontal | NA | NA | NA | 4.547 | 5.66E-05 | 0.0017 |
| R_rostralmiddlefrontal | NA | NA | NA | 3.233 | 2.58E-03 | 0.0167 |
| L_superiorfrontal | NA | NA | NA | 6.029 | 5.73E-07 | 0.00006 |
| R_superiorfrontal | NA | NA | NA | 5.395 | 4.14E-06 | 0.0002 |
| L_superiorparietal | NA | NA | NA | 2.663 | 0.0114 | 0.0484 |
| R_superiorparietal | NA | NA | NA | 2.425 | 0.0203 | 0.0768^c^ |
| L_superiortemporal | NA | NA | NA | 3.304 | 0.0021 | 0.0159 |
| R_superiortemporal | NA | NA | NA | 3.417 | 0.0016 | 0.0132 |
| L_supramarginal | NA | NA | NA | 3.825 | 0.0005 | 0.0060 |
| R_supramarginal | NA | NA | NA | 3.274 | 0.0023 | 0.0159 |
| L_caudalanteriorcingulate | NA | NA | NA | 2.682 | 0.0109 | 0.0484 |
| L_fusiform | NA | NA | NA | 2.758 | 0.0090 | 0.0436 |
| L_inferiorparietal | NA | NA | NA | 3.527 | 0.0011 | 0.0104 |
| L_medialorbitofrontal | NA | NA | NA | 3.566 | 0.0010 | 0.0099 |
| L_middletemporal | NA | NA | NA | 4.313 | 0.0001 | 0.0026 |
| L_precentral | NA | NA | NA | 3.270 | 0.0023 | 0.0159 |
| R_ bankssts | NA | NA | NA | 2.695 | 0.0105 | 0.0484 |
| R_ posteriorcingulate | NA | NA | NA | 3.076 | 0.0039 | 0.0243 |
| R_ precuneus | NA | NA | NA | 2.673 | 0.0111 | 0.0484 |
| R_ insula | NA | NA | NA | 3.056 | 0.0041 | 0.0245 |

Note: Four patients withdrew the follow-up scans. ^a^Patients_0W (n = 42) *vs.* Healthy controls (n = 38), two-sample *t* test; ^b^Patients_0W (n=38) *vs.* Patients_8W (n = 38), paired-sample *t* test; ^c^marginal significance. Abrreviations: NA = not applicable; L = Left; R = Right.

**Table S4** The TOP 20 enriched pathways and biological processes of the PLS1 genes examined in the spatial correlation analysis of the treatment-related paired t-statistic map and Allen genes expression

| **Terms** | **Category（GO/KEGG）** | **Description** | **Count** | **%** | **Log10(*P*)** | **Log10(*q*)** |
| --- | --- | --- | --- | --- | --- | --- |
| GO:0050804 | GO Biological Processes | modulation of chemical synaptic transmission | 87 | 4.15 | -19.17 | -15.20 |
| GO:0031344 | GO Biological Processes | regulation of cell projection organization | 108 | 5.16 | -17.31 | -13.59 |
| hsa05200 | KEGG Pathway | Pathways in cancer | 91 | 4.34 | -15.05 | -11.55 |
| GO:0035239 | GO Biological Processes | tube morphogenesis | 100 | 4.77 | -12.89 | -9.54 |
| GO:0007610 | GO Biological Processes | behavior | 89 | 4.25 | -12.63 | -9.33 |
| GO:0006468 | GO Biological Processes | protein phosphorylation | 103 | 4.92 | -12.49 | -9.25 |
| GO:0034330 | GO Biological Processes | cell junction organization | 78 | 3.72 | -12.10 | -8.90 |
| GO:0051046 | GO Biological Processes | regulation of secretion | 92 | 4.39 | -12.00 | -8.84 |
| GO:0043549 | GO Biological Processes | regulation of kinase activity | 107 | 5.11 | -11.61 | -8.49 |
| GO:0046903 | GO Biological Processes | secretion | 78 | 3.72 | -11.53 | -8.45 |
| GO:0051668 | GO Biological Processes | localization within membrane | 81 | 3.87 | -11.43 | -8.39 |
| GO:0034097 | GO Biological Processes | response to cytokine | 110 | 5.25 | -11.41 | -8.39 |
| GO:0007423 | GO Biological Processes | sensory organ development | 84 | 4.01 | -11.10 | -8.11 |
| GO:0098609 | GO Biological Processes | cell-cell adhesion | 81 | 3.87 | -10.66 | -7.69 |
| GO:0043269 | GO Biological Processes | regulation of ion transport | 96 | 4.58 | -10.46 | -7.57 |
| hsa04024 | KEGG Pathway | cAMP signaling pathway | 45 | 2.15 | -10.27 | -7.46 |
| hsa05032 | KEGG Pathway | Morphine addiction | 27 | 1.29 | -10.27 | -7.46 |
| GO:0030001 | GO Biological Processes | metal ion transport | 88 | 4.20 | -10.26 | -7.46 |
| GO:0060627 | GO Biological Processes | regulation of vesicle-mediated transport | 79 | 3.77 | -10.04 | -7.27 |
| GO:0030335 | GO Biological Processes | positive regulation of cell migration | 82 | 3.91 | -9.96 | -7.23 |

Abbreviations: GO, gene ontology; KEGG, Kyoto encyclopedia of genes and genomes.

**References:**

1. Johnson WE, Li C, Rabinovic A. Adjusting batch effects in microarray expression data using empirical Bayes methods. *Biostatistics.* (2007) 8:118-27. doi: 10.1093/biostatistics/kxj037.

2. Horvath S. DNA methylation age of human tissues and cell types. *Genome Biol.* (2013) 14:R115. doi: 10.1186/gb-2013-14-10-r115.
